# Supplementary material for: Molecularly defined and spatially resolved cell atlas of the whole mouse brain
Source: Nature. 2023 Dec 13;624(7991):343–54. doi: 10.1038/s41586-023-06808-9 (PMC10719103; doi:10.1038/s41586-023-06808-9)
Supplement: Supplementary file 1 — Reporting Summary [file 41586_2023_6808_MOESM1_ESM.pdf]

nature portfolio

Corresponding author(s): Xiaowei Zhuang

Last updated by author(s): Oct 29, 2023

Reporting Summary

Nature Portfolio wishes to improve the reproducibility of the work that we publish. This form provides structure for consistency and transparency in reporting. For further information on Nature Portfolio policies, see our Editorial Policies and the Editorial Policy Checklist.

Statistics

For all statistical analyses, confirm that the following items are present in the figure legend, table legend, main text, or Methods section.

n/a	Confirmed	
☐	☒	The exact sample size (n) for each experimental group/condition, given as a discrete number and unit of measurement
☐	☒	A statement on whether measurements were taken from distinct samples or whether the same sample was measured repeatedly
☐	☒	The statistical test(s) used AND whether they are one- or two-sided<br>*Only common tests should be described solely by name; describe more complex techniques in the Methods section.*
☒	☐	A description of all covariates tested
☐	☒	A description of any assumptions or corrections, such as tests of normality and adjustment for multiple comparisons
☐	☒	A full description of the statistical parameters including central tendency (e.g. means) or other basic estimates (e.g. regression coefficient) AND variation (e.g. standard deviation) or associated estimates of uncertainty (e.g. confidence intervals)
☐	☒	For null hypothesis testing, the test statistic (e.g. F, t, r) with confidence intervals, effect sizes, degrees of freedom and P value noted<br>*Give P values as exact values whenever suitable.*
☒	☐	For Bayesian analysis, information on the choice of priors and Markov chain Monte Carlo settings
☒	☐	For hierarchical and complex designs, identification of the appropriate level for tests and full reporting of outcomes
☐	☒	Estimates of effect sizes (e.g. Cohen's d, Pearson's r), indicating how they were calculated

Our web collection on statistics for biologists contains articles on many of the points above.

Software and code

Policy information about availability of computer code

Data collection	MERFISH imaging data was collected using custom Python code to control the microscope. This code is available at https://github.com/ZhuangLab and on Zenodo.
Data analysis	The MERFISH data was analyzed using custom Python code. Code for MERFISH image analysis is available at https://github.com/ZhuangLab/MERlin and on Zenodo. Additional code for data analysis is available at https://github.com/ZhuangLab/whole_mouse_brain_MERFISH_atlas_scripts_2023 and on Zenodo.<br>Other packages used in data analyses include: Cellpose (version 2.0); Scanpy (version 1.9.1); Scrublet (version 0.2); ALLCools (version 0.2.19); metis (version 0.2a5); scikit-learn (version 1.1.1); Elastix (version 5.1.0); Brainrender (version 2.0.0.0).

For manuscripts utilizing custom algorithms or software that are central to the research but not yet described in published literature, software must be made available to editors and reviewers. We strongly encourage code deposition in a community repository (e.g. GitHub). See the Nature Portfolio guidelines for submitting code & software for further information.

Data

Policy information about availability of data

All manuscripts must include a data availability statement. This statement should provide the following information, where applicable:

- Accession codes, unique identifiers, or web links for publicly available datasets
- A description of any restrictions on data availability
- For clinical datasets or third party data, please ensure that the statement adheres to our policy

Data availability statement is included in the manuscript, which states:

Raw and processed MERFISH data, as well as the MERFISH codebook and probes used in this work, can be accessed via the Brain Image Library (BIL): https://doi.org/10.35077/act-bag . Processed MERFISH data are also accessible and explorable in an interactive manner through two platforms: 1. Allen Brain Cell (ABC) Atlas (https://knowledge.brain-map.org/data/5C0201JSVE04WY6DMVC/explore; https://allen-brain-cell-atlas.s3.us-west-2.amazonaws.com/index.html); 2. CELLxGENE database (https://cellxgene.cziscience.com/collections/0cca8620-8dee-45d0-aef5-23f032a5cf09).

The scRNA-seq datasets (FASTQ files) obtained by Allen Institute are available at NeMO under identifier https://assets.nemoarchive.org/dat-qg7n1b0. The processed scRNA-seq data along with the transcriptomic cell type taxonomy is visualized at ABC Atlas – mouse whole brain cell type atlas, https://portal.brain-map.org/atlases-and-data/bkp/abc-atlas. Instruction for access of the processed scRNA-seq data is available at https://github.com/AllenInstitute/abc_atlas_access/blob/main/descriptions/WMB-10X.md.

CellChat database is available at (http://www.cellchat.org/).

Human research participants

Policy information about studies involving human research participants and Sex and Gender in Research.

Reporting on sex and gender	Not applicable
Population characteristics	Not applicable
Recruitment	Not applicable
Ethics oversight	Not applicable

Note that full information on the approval of the study protocol must also be provided in the manuscript.

Field-specific reporting

Please select the one below that is the best fit for your research. If you are not sure, read the appropriate sections before making your selection.

☒ Life sciences ☐ Behavioural & social sciences ☐ Ecological, evolutionary & environmental sciences

For a reference copy of the document with all sections, see nature.com/documents/nr-reporting-summary-flat.pdf

Life sciences study design

All studies must disclose on these points even when the disclosure is negative.

Sample size	Four replicate mice, one female and three males, were imaged under each condition. From the four replicate mice imaged for the identification and spatial mapping of cell types, a total of approximately 10 million cells were imaged and segmented, which generated a sufficient number of single-cell profiles and gave sufficient statistics for the effect sizes of interest. No statistical methods were used to predetermine sample size and sample size were determined empirically.
Data exclusions	We did not exclude any data from consideration. All images were included in the primary analysis.
Replication	Reported results were replicated from four animals under each condition.
Randomization	Four animals, one female and three males, were randomly chosen for the identification and spatial mapping of cell types. For each mouse, the imaging experiments were definitive, and no randomization was necessary for this study, hence the experiments were not randomized. Animals were not allocated into experimental groups.
Blinding	The investigators were not blinded during experiments and outcome assessment. Blinding during data collection was not needed because all images were taken under same condition. Blinding during analysis was not necessary because the results were quantitative and did not require subjective judgment. Blinding is not typically used in the field.

Reporting for specific materials, systems and methods

We require information from authors about some types of materials, experimental systems and methods used in many studies. Here, indicate whether each material, system or method listed is relevant to your study. If you are not sure if a list item applies to your research, read the appropriate section before selecting a response.

Materials & experimental systems

n/a	Involved in the study
☒	☐ Antibodies
☒	☐ Eukaryotic cell lines
☒	☐ Palaeontology and archaeology
☐	☒ Animals and other organisms
☒	☐ Clinical data
☒	☐ Dual use research of concern

Methods

n/a	Involved in the study
☒	☐ ChIP-seq
☒	☐ Flow cytometry
☒	☐ MRI-based neuroimaging

Animals and other research organisms

Policy information about studies involving animals; ARRIVE guidelines recommended for reporting animal research, and Sex and Gender in Research

Laboratory animals	Adult C57BL/6NCrl (Strain code: 027, Charles River Laboratories) male and female mice aged 56-62 days were used in this study. Animals were purchased from the Charles River Laboratories at an age one week younger (49-55 days) than the target age for sacrifice and housed at Harvard University Animal Facility for 1 week to acclimate before sacrifice. Mice were maintained on a 12 hour:12 hour light/dark cycle (2pm-2am dark period) with at a temperature of 22 ± 1°C, a humidity of 30–70%, with ad libitum access to food and water. All the animals used in this study were sacrificed between 2-6pm of the day.
Wild animals	The study did not involve wild animals.
Reporting on sex	Four animals were used in this study, including one female mice and three male mice. No sex-specific results are reported.
Field-collected samples	The study did not involve samples collected from the field.
Ethics oversight	Harvard University Institutional Animal Care and Use Committee

Note that full information on the approval of the study protocol must also be provided in the manuscript.
